# Supplementary material for: Unravelling cytosolic delivery of cell penetrating peptides with a quantitative endosomal escape assay
Source: Nat Commun. 2021 Jun 17;12:3721. doi: 10.1038/s41467-021-23997-x (PMC8211857; doi:10.1038/s41467-021-23997-x)
Supplement: Supplementary file 1 — Supplementary information [file 41467_2021_23997_MOESM1_ESM.pdf]

Supplementary Information

Unravelling cytosolic delivery of cell penetrating peptides with a quantitative endosomal escape assay

Serena L.Y. Teo[1,2†], Joshua J. Rennick[1,2†], Daniel Yuen[1,2], Hareth Al-Wassiti[1], Angus P.R. Johnston[1,2*], Colin W. Pouton[1*]

[1]Monash Institute of Pharmaceutical Sciences, Monash University, Parkville, Victoria, Australia.

[2]ARC Centre of Excellence in Convergent Bio-Nano Science and Technology, Monash University, Parkville, Australia.

† These authors contributed equally to the work

* Correspondence

Email: angus.johnston@monash.edu, colin.pouton@monash.edu

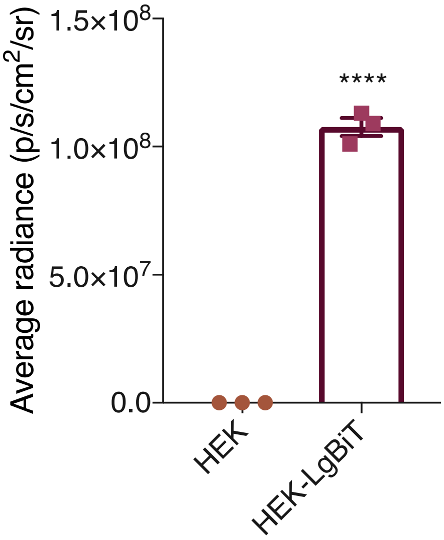

Supplementary Fig. 1: Successful expression of LgBiT protein in HEK293 cells. Non-transduced HEK293 cells and HEK293-LgBiT cells were seeded at 10,000 cells/well in black 96-well clear bottom plates. After overnight incubation, cells were treated with 1 nM HiBiT peptide and 0.01% w/v digitonin for 1 hour at 37ºC to completely permeabilise the cells. NanoGlo Live Cell substrate was added to cells and luminescence was measured on IVIS Lumina II. Data represents mean ± SEM, n=3 independent experiments. **** denotes p ≤ 0.0001

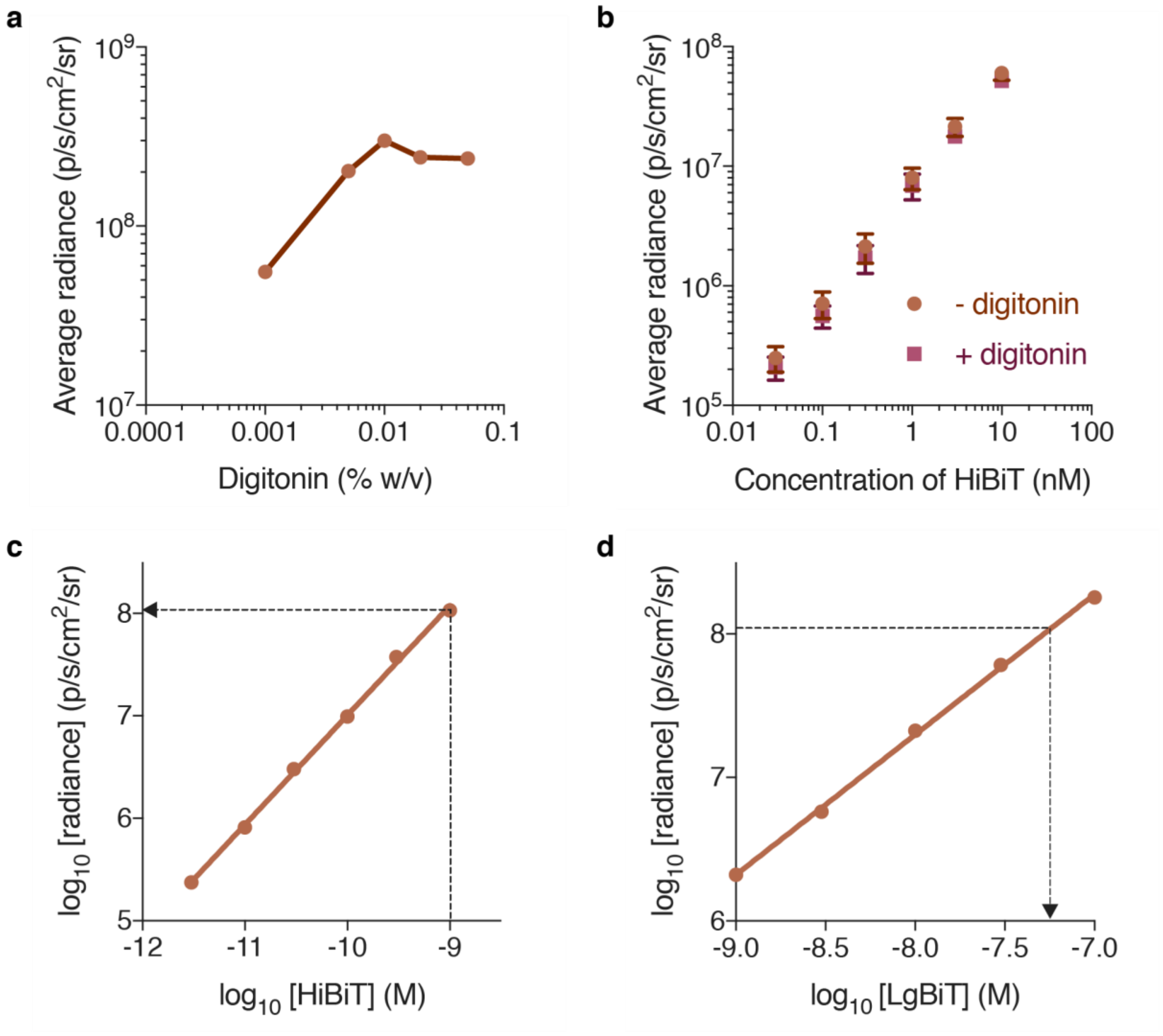

Supplementary Fig. 2: Concentration of LSA expressed in HEK293-LSA cells. a) 0.01% wt/v digitonin is sufficient to achieve total cell permeabilization. HEK293-LSA cells were seeded at 10,000 cells/well in black 96-well plates. After overnight incubation, cells were incubated with 1 µM HiBiT peptide for 2 hours. Cells were washed and treated with various concentrations of digitonin (0.001, 0.005, 0.01, 0.02 and 0.05% w/v) for 30 minutes. NanoGlo Live Cell substrate was added and luminescence was measured on PerkinElmer In Vivo Imaging System Lumina II (IVIS) 30 minutes after substrate addition. Data represents mean, n =2 independent experiments. b) Digitonin does not affect luciferase activity. Purified LgBiT (50 nM) was combined with various concentrations of HiBiT peptide (0.03, 0.1, 0.3, 1, 3, 10 nM) with or without the presence of 0.01% wt/v digitonin. No difference in emitted radiance was observed with or without digitonin. Data represents mean ± SD, n=3 independent experiments. c) Linear luminescence in permeabilised HEK293-LSA cells was obtained over concentration range of 3 pM to 1 nM HiBiT. HEK293-LSA cells seeded in black 96-well clear bottom microplates at 10,000 cells/well were permeabilised with digitonin (0.01% wt/v) and treated with various concentrations of HiBiT peptide. Linear regression analysis was used to determine the line of best fit. Data represents mean ± SD, n=3. d) Total concentration of LSA expressed in HEK293 cells is determined to be 55.8 nM. Fixed 1 nM HiBiT peptide was titrated with various concentrations of purified LgBiT protein to construct a calibration curve. Data represents mean ± SD, n=2. The y-value at 1 nM HiBiT peptide in Fig. S2c (see arrow in Fig. S2c, y = 8.03) was interpolated to obtain the $\log_{10}$

[LgBiT] value in the curve obtained (see arrow in Fig. S2d, x = -7.25). The estimated concentration of LSA expressed in cells is therefore $10^{-7.253}$ = 55.8 nM.

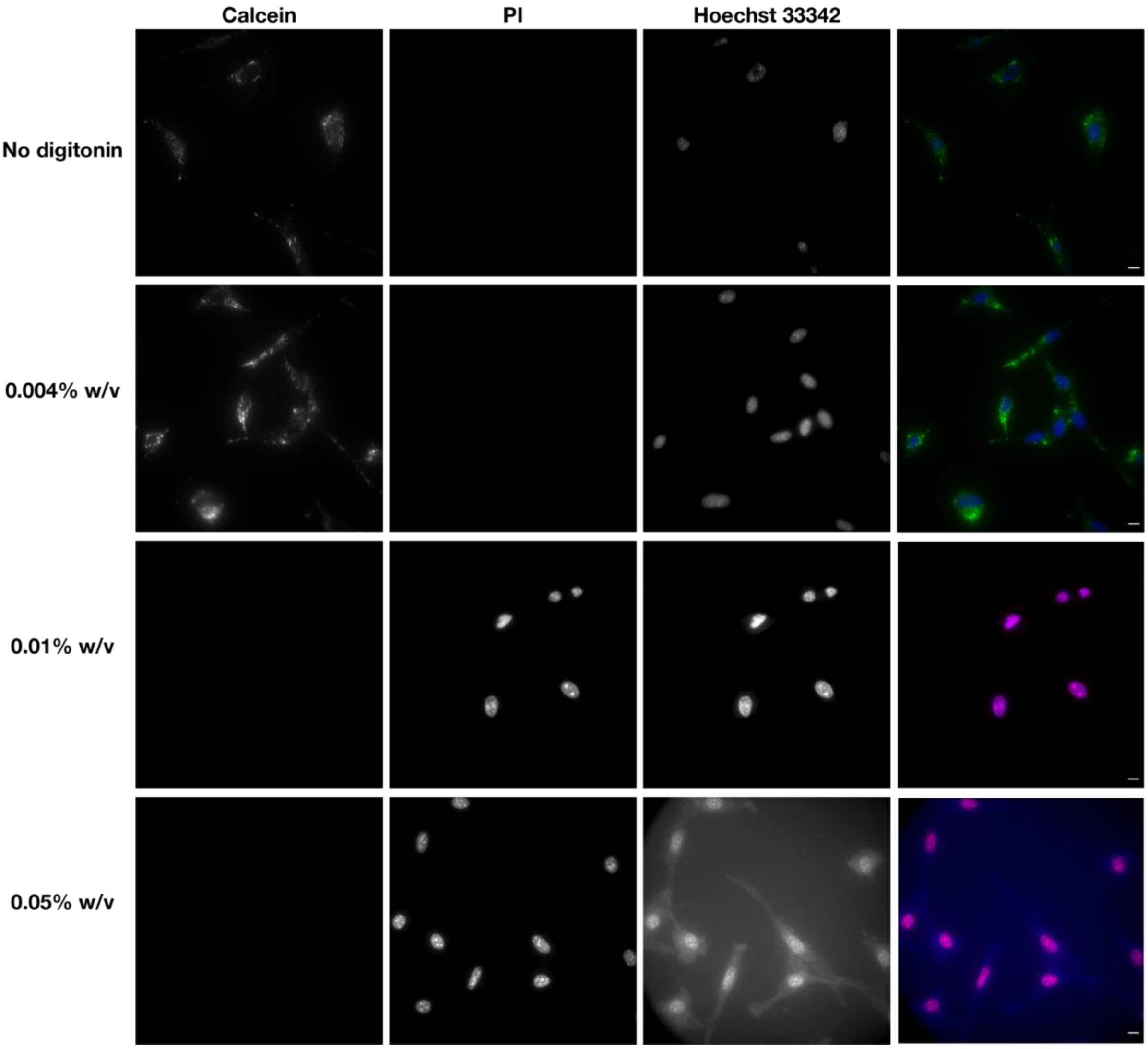

Supplementary Fig. 3: Complete cell permeablisation can be achieved with 0.01% w/v digitonin. 3T3 cells were incubated with 100 µg/mL calcein for 2 hours prior to treatment with increasing concentrations of digitonin. Cells were then stained with Hoescht 33342 and propidium iodide (PI). Hoechst 33342 stains all cell nuclei (blue) and PI only stains nuclei of permeabilised cells (red). Calcein (green) is used as an endosomal marker. Complete permeabilisation of membranes is achieved at 0.01% w/v digitonin, shown by the presence of PI fluorescence and loss of calcein fluorescence. n=2 independent experiments. Scale bar = 10 µm

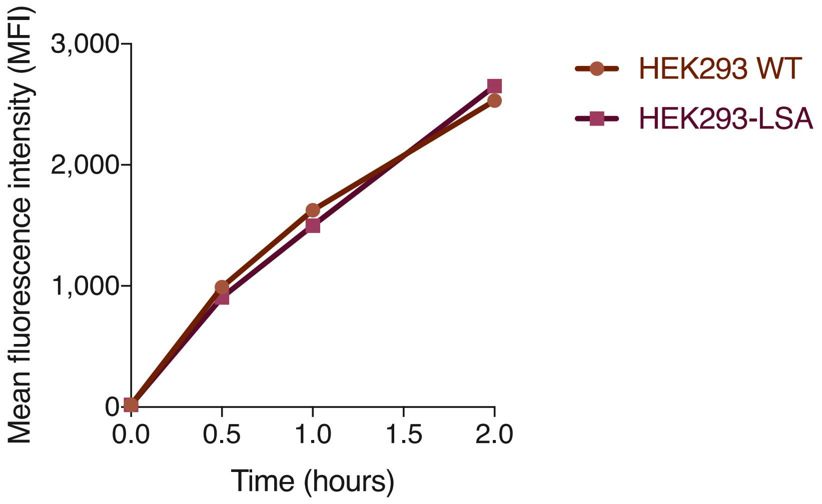

Supplementary Fig. 4: Wild type (WT) HEK293 and HEK293-LSA cells show similar calcein uptake. HEK293 (WT) and HEK293-LSA cells were seeded at 40,000 cells/well in a 96-well plate. After overnight incubation, the cells were treated with calcein for 0.5, 1 and 2 hours respectively. The cells were washed and then analysed by flow cytometry. n=1 experiment

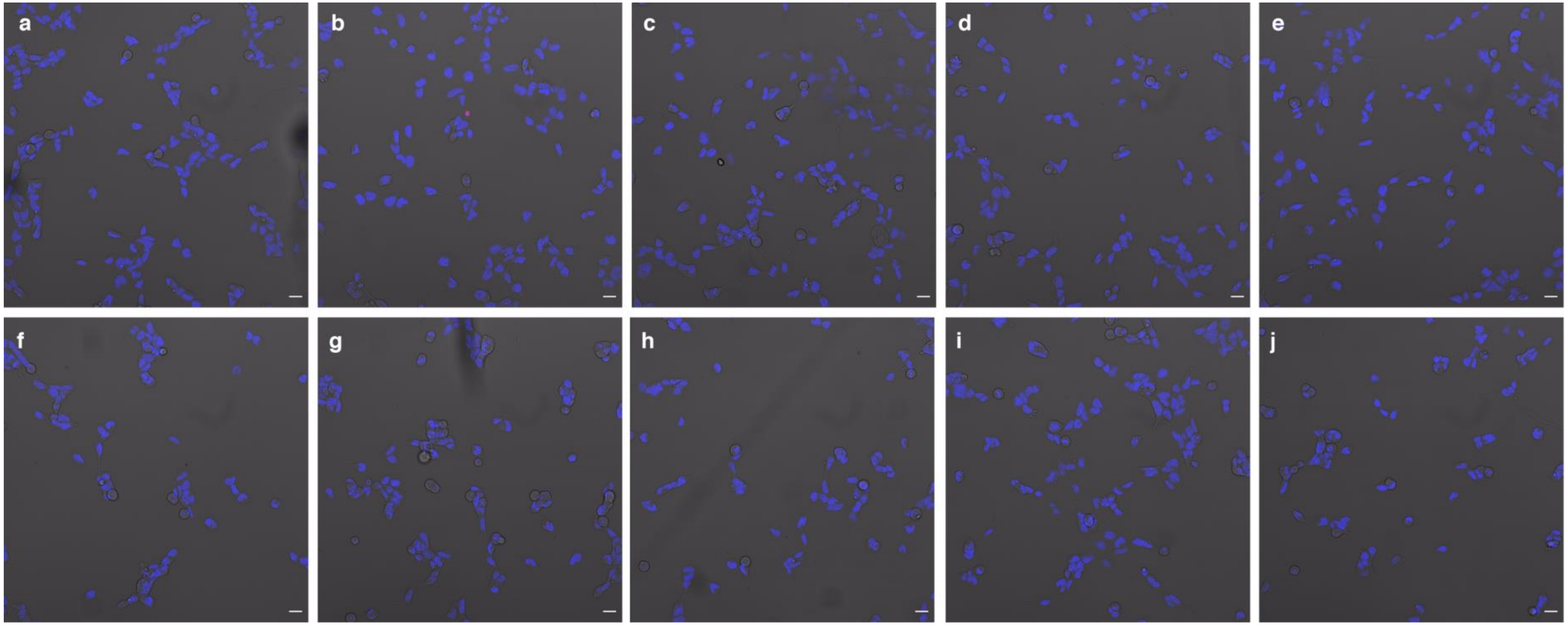

Supplementary Fig. 5: HEK293-LSA cells maintain viability after treatment with EEP-GFP-HiBiT proteins. After a 4-hour incubation with 1 µM EEP-GFP-HiBiT proteins or free HiBiT peptide: a) GFP, b) R9, c) TAT, d) ZF5.3, e) E5T**A**T, f) 5.3, g) pHD118, h) pHlip, i) HA2 and j) HiBiT, HEK293-LSA cells were washed and stained with Hoechst 33342 and propidium iodide (PI). Cells were imaged with confocal microscope. Hoechst 33342 stains all cell nuclei (blue) and PI only stains nuclei of dead cells (red). Fluorescence images are overlaid on bright-field image. Cells displayed their typical morphology, and less than 1% of the cells were positive for PI. n=2 independent experiments. Scale bar = 10 µm

a

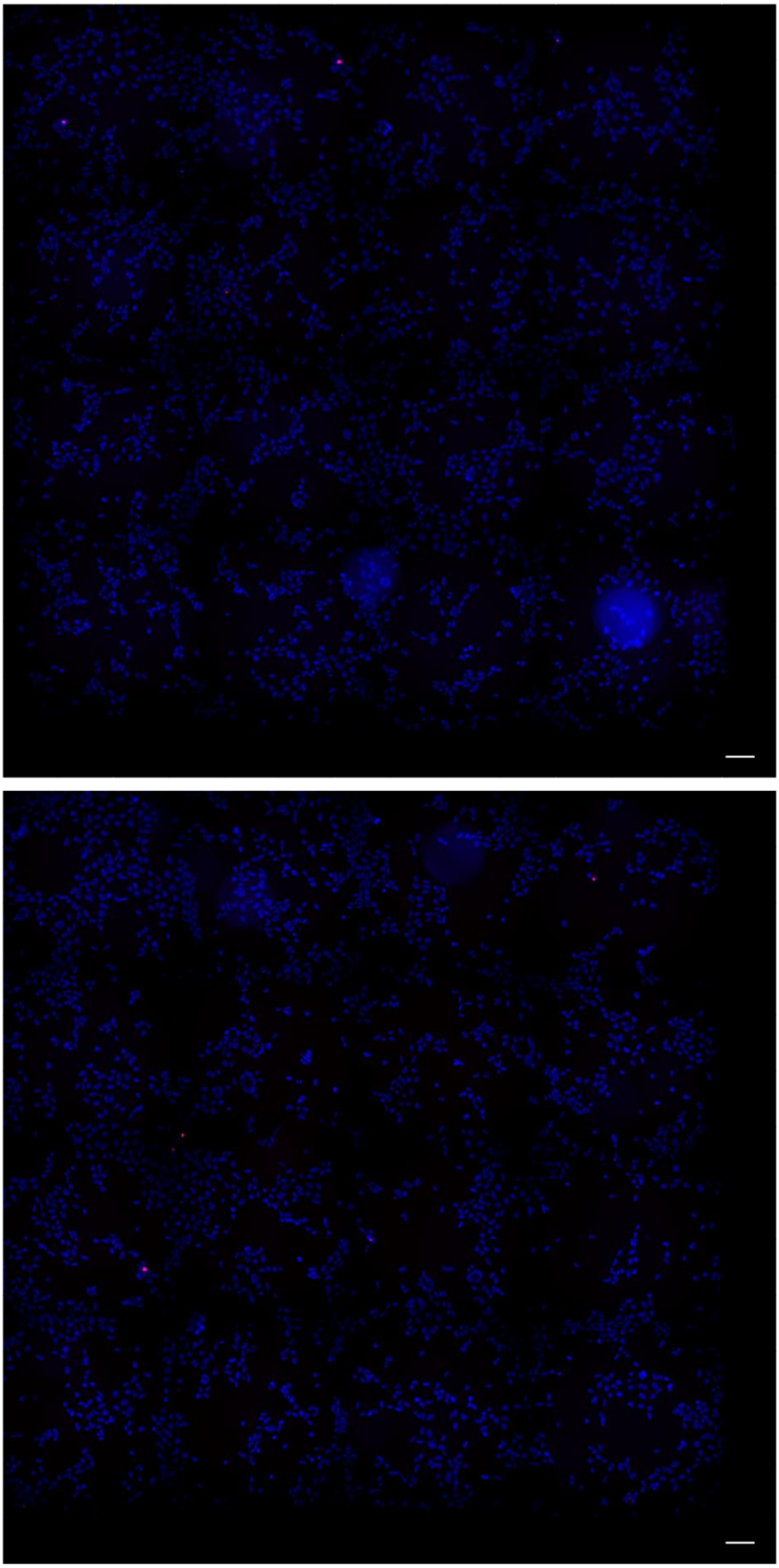

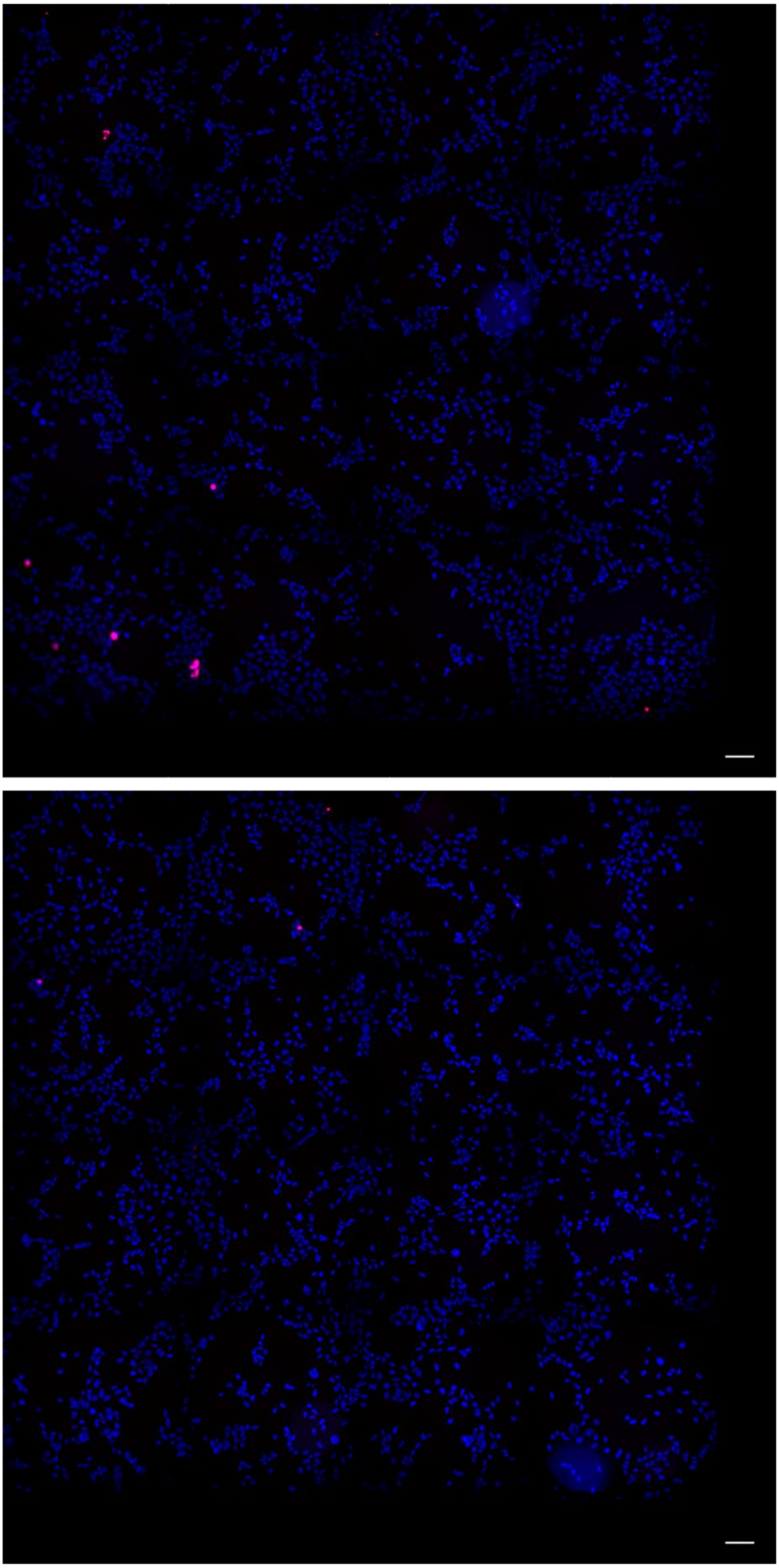

b

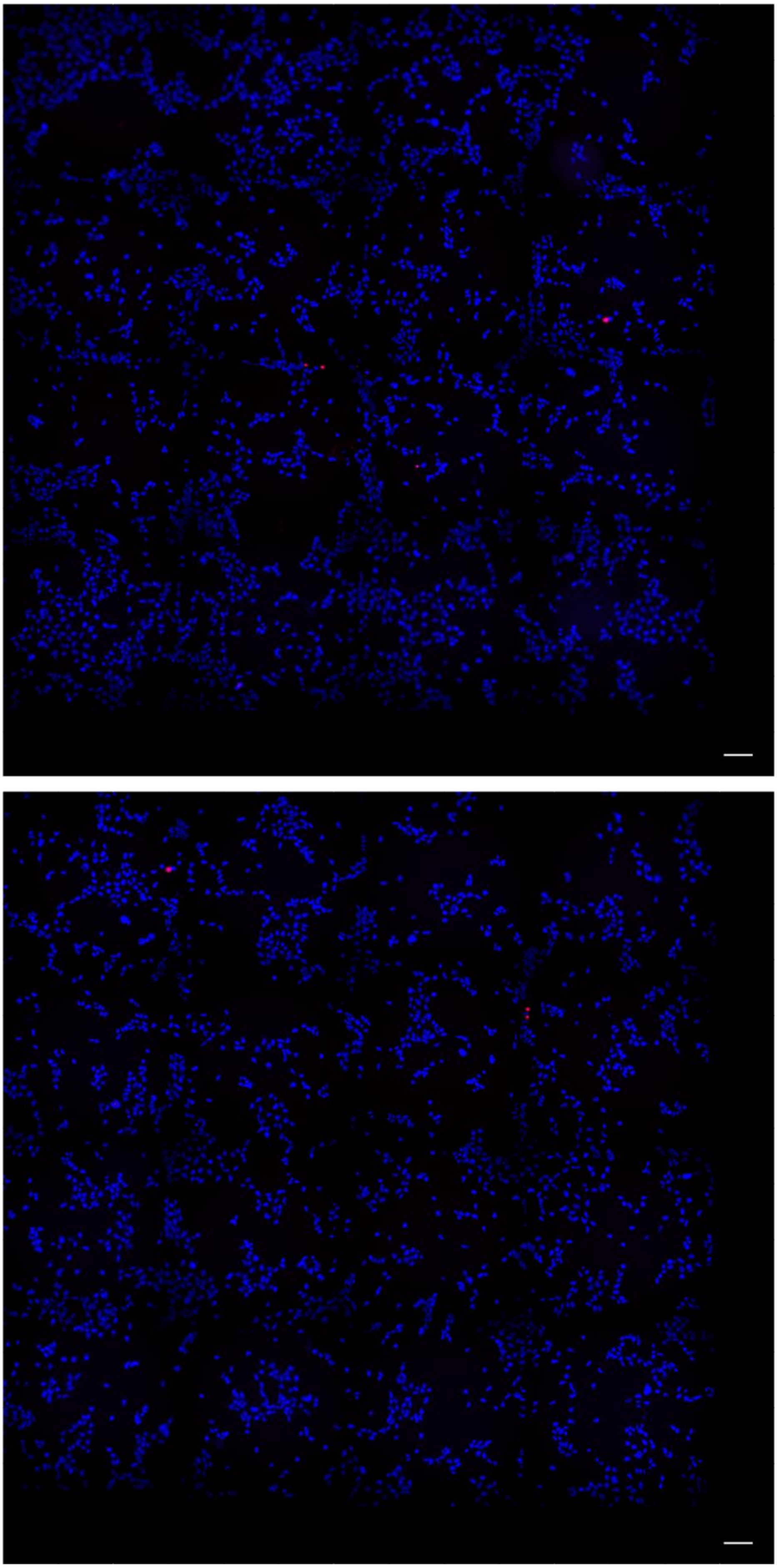

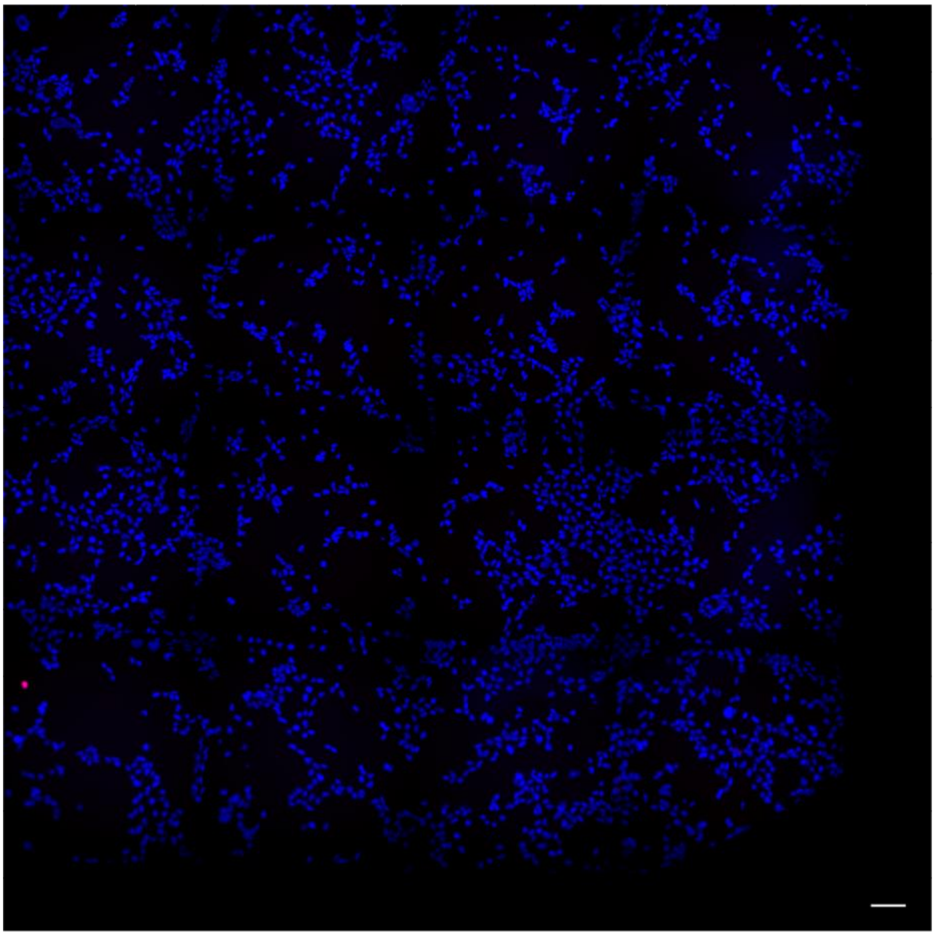

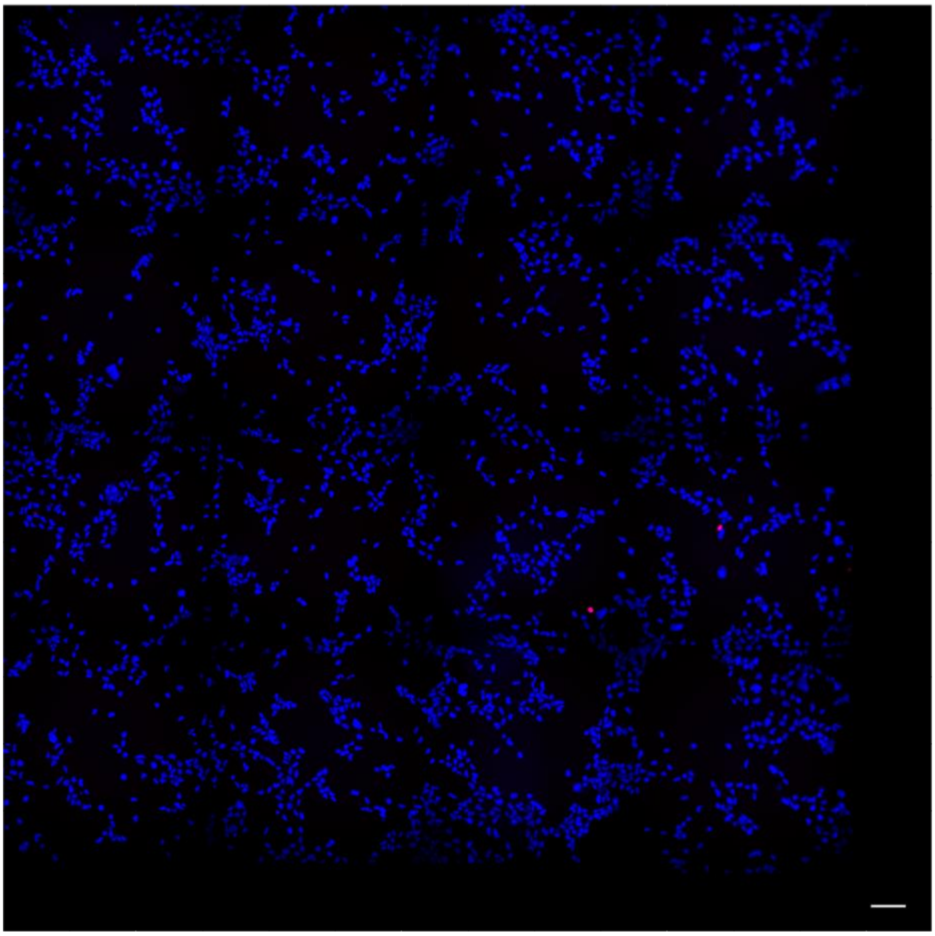

Supplementary Fig. 6: HEK293-LSA cells maintain viability after treatment with GFP-HiBiT. Montage images of a) untreated HEK293-LSA and b) cells treated with 1 µM GFP-HiBiT. After 4-hour incubation, cells were washed and stained with Hoechst 33342 and propidium iodide (PI). Hoechst 33342 stains all cell nuclei (blue) and PI only stains nuclei of dead cells (red). Less than 1% of the cells were positive for PI. n=2 independent experiments. Scale bar = 100 µm

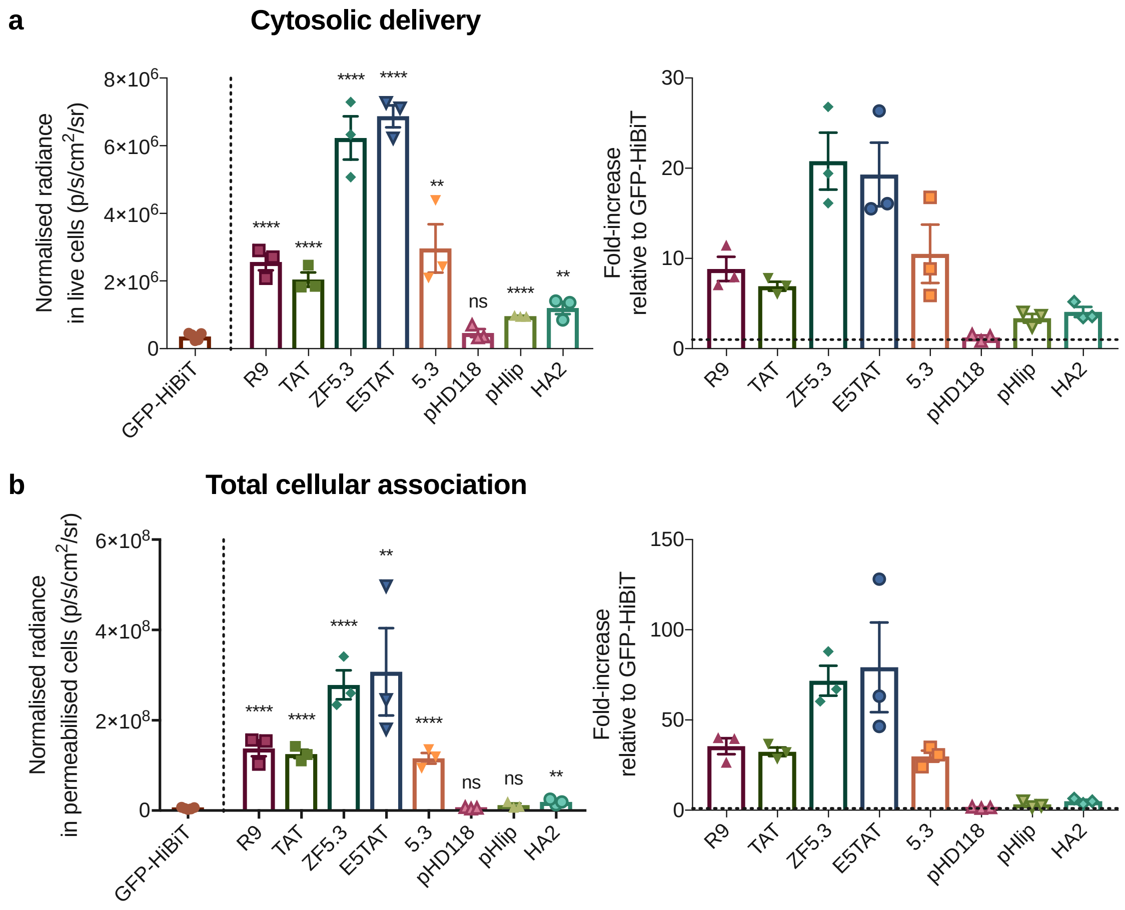

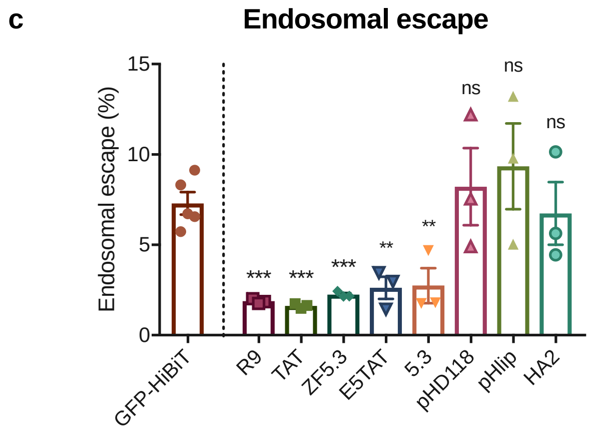

d

Protein	Cytosolic luminescence (x10^{-6}) (p/s/cm^2/sr)	Total cellular association luminescence (x10^{-6}) (p/s/cm^2/sr)	Endosomal escape efficiency (%)
GFP	0.36 ± 0.05	5.16 ± 0.85	7.29 ± 0.62
R9	2.56 ± 0.25	138 ± 17	1.88 ± 0.08
TAT	2.04 ± 0.21	125 ± 9	1.62 ± 0.07
ZF5.3	6.23 ± 0.64	278 ± 32	2.25 ± 0.09
E5TAT	6.87 ± 0.33	307 ± 97	2.62 ± 0.61
5.3	2.96 ± 0.71	116 ± 12	2.74 ± 0.97
pHD118	0.46 ± 0.12	5.78 ± 0.84	8.22 ± 2.14
pHlip	0.97 ± 0.01	12.2 ± 3.8	9.35 ± 2.37
HA2	1.21 ± 0.18	19.2 ± 3.4	6.74 ± 1.74

Supplementary Fig. 7: Cationic EEPs increase cytosolic delivery of GFP but do not increase endosomal escape efficiency in HeLa cells. a) Cytosolic luminescent signal of EEP-GFP-HiBiT in HeLa-LSA cells and fold-increase in signal with respect to GFP (represented by dotted line = 1). HeLa-LSA cells were incubated with EEP-GFP-HiBiT proteins at 1 µM for 4 hours. Data represents mean ± SEM, n=3 independent experiments. Kruskal-Wallis with uncorrected Dunn's test was used to analyse the data. b) Total cellular association of EEP-GFP-HiBiT in HeLa cells and fold-increase with respect to GFP (represented by dotted line = 1) determined by permeabilising the cells using 0.01% w/v digitonin. Data represents mean ± SEM, n=3 independent experiments. Kruskal-Wallis with uncorrected Dunn's test was used to analyse the data. c) Endosomal escape efficiency of EEP-GFP-HiBiT proteins determined by ratioing cytosolic signal with total cellular association. Data represents mean ± SEM, n=3 independent experiments. Kruskal-Wallis with uncorrected Dunn's test was used to analyse the data.d) Summary of cytosolic luminescence, total cellular association luminescence and endosomal escape efficiency for all proteins. Data represents mean ± SEM. Data represents mean ± SEM, n=3 independent experiments. Kruskal-Wallis with uncorrected Dunn's test was used to analyse the data, with GFP being the control group. ns denote $p > 0.05$, * denotes $p < 0.05$, ** denotes $p < 0.01$ and *** denotes $p < 0.001$

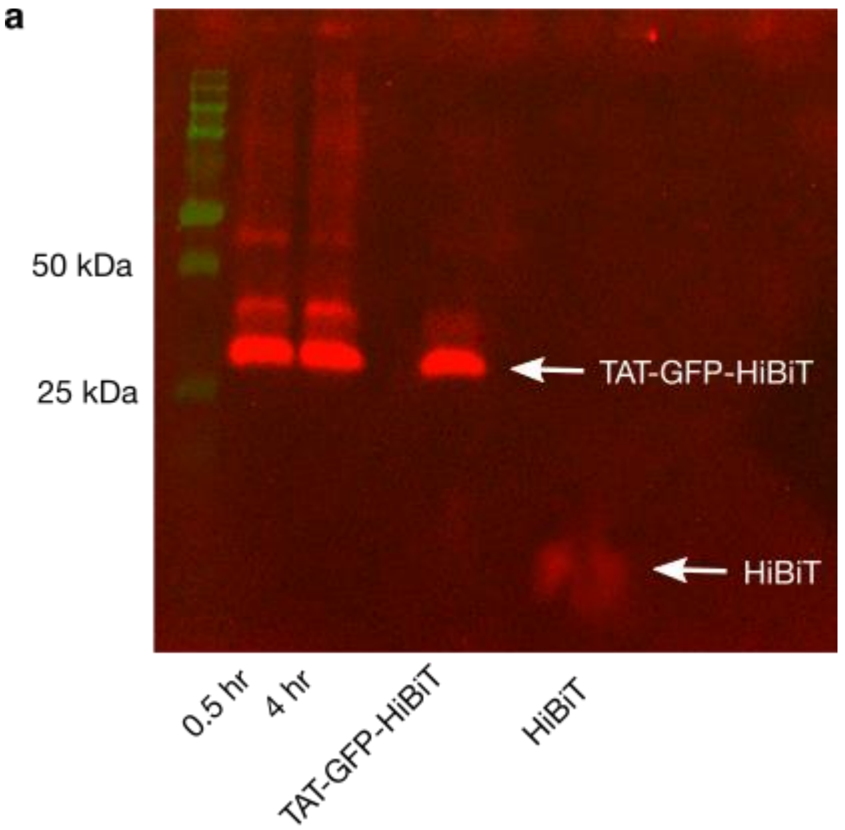

b

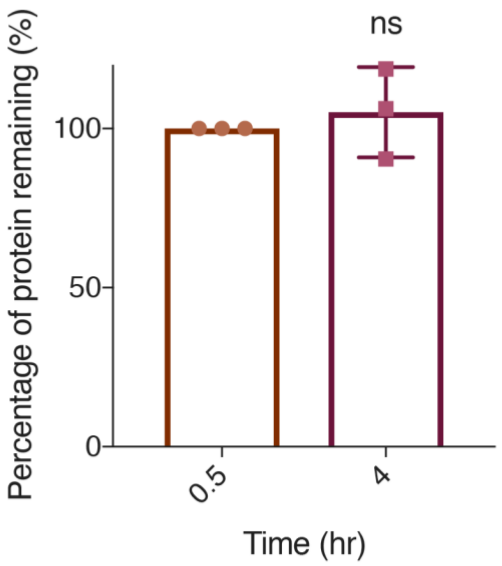

Supplementary Fig. 8: TAT-GFP-HiBiT protein does not degrade after 4-hour incubation in HEK293 cells. a) TAT-GFP-HiBiT protein (1 µM) was allowed to bind to surface of HEK293 cells for 1 hour on ice. Unbound proteins were washed away and cells were incubated at 37ºC for 0.5 or 4 hours to allow internalisation to take place. Cells were lysed with RIPA buffer (supplemented with protease inhibitor) and lysate was analysed by western blot. Untreated cell lysate was spiked with TAT-GFP-HiBiT or free HiBiT peptide. n = 3 independent experiments b) Percentage of intact TAT-GFP-HiBiT remaining in cell lysate was quantified using densitometric analysis of western blot. No significant decrease in the amount of intact TAT-GFP-HiBiT was observed after 4 hours. Two-tailed unpaired t test was used to analyse the data. Data represents mean ± SEM, n = 3 independent experiments. ns (not significant) denotes $p > 0.05$

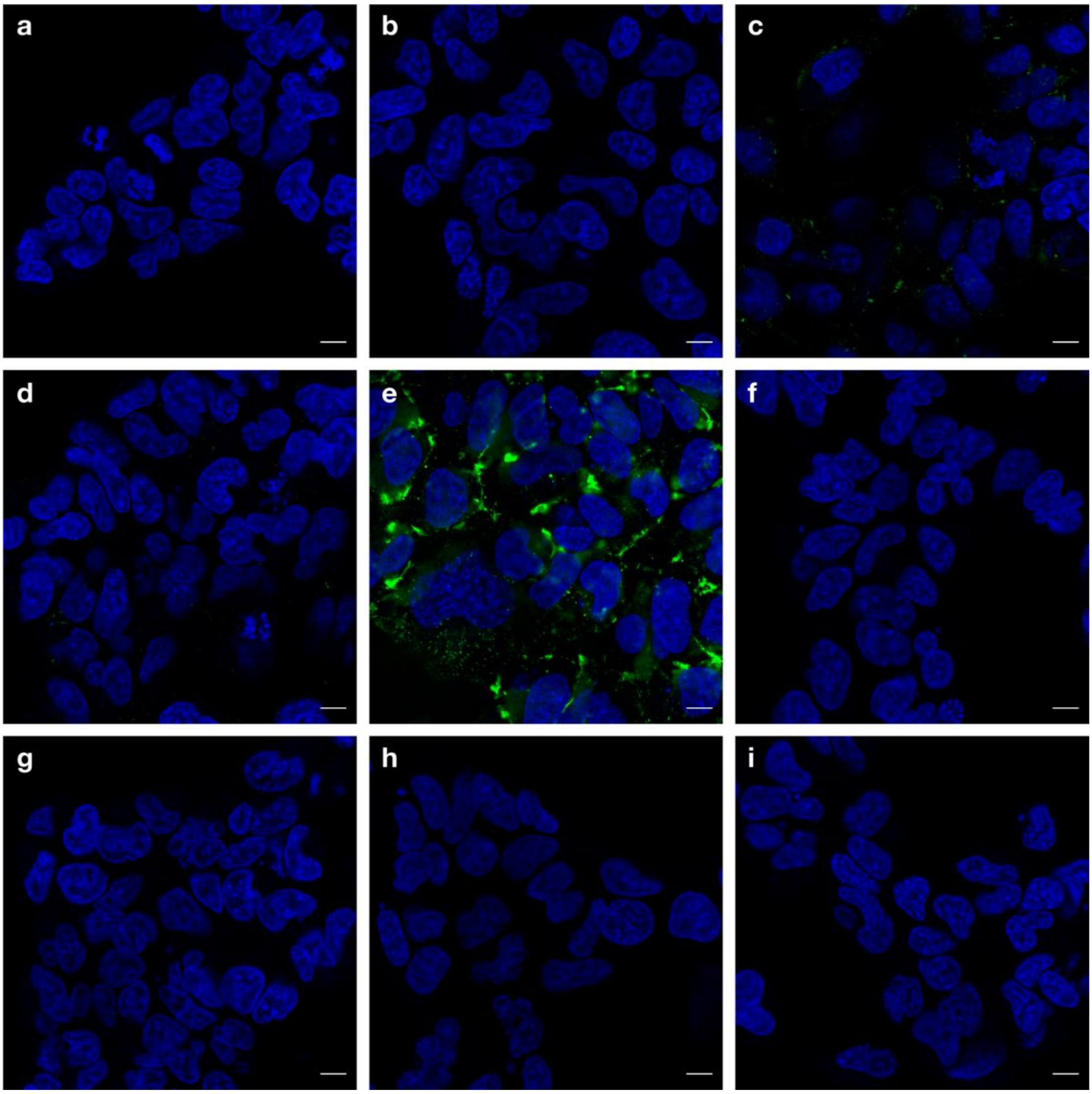

Supplementary Fig. 9: EEP-GFP-HiBiT fusion proteins exhibit punctate staining, suggesting limited endosomal escape. HEK293-LSA cells treated with EEP-GFP-HiBiT (green) proteins: a) GFP, b) R9, c) TAT, d) ZF5.3, e) E5TAT, f) 5.3, g) pHD118, h) pHlip and i) HA2 at 1 µM for 4 hours. Cells were washed and nuclei was stained with Hoechst 33342 (blue) before confocal microscopy. Images displayed with the same dynamic range to enable comparison of intensity. n=2 independent experiments. Scale bar = 10 µm

a

b

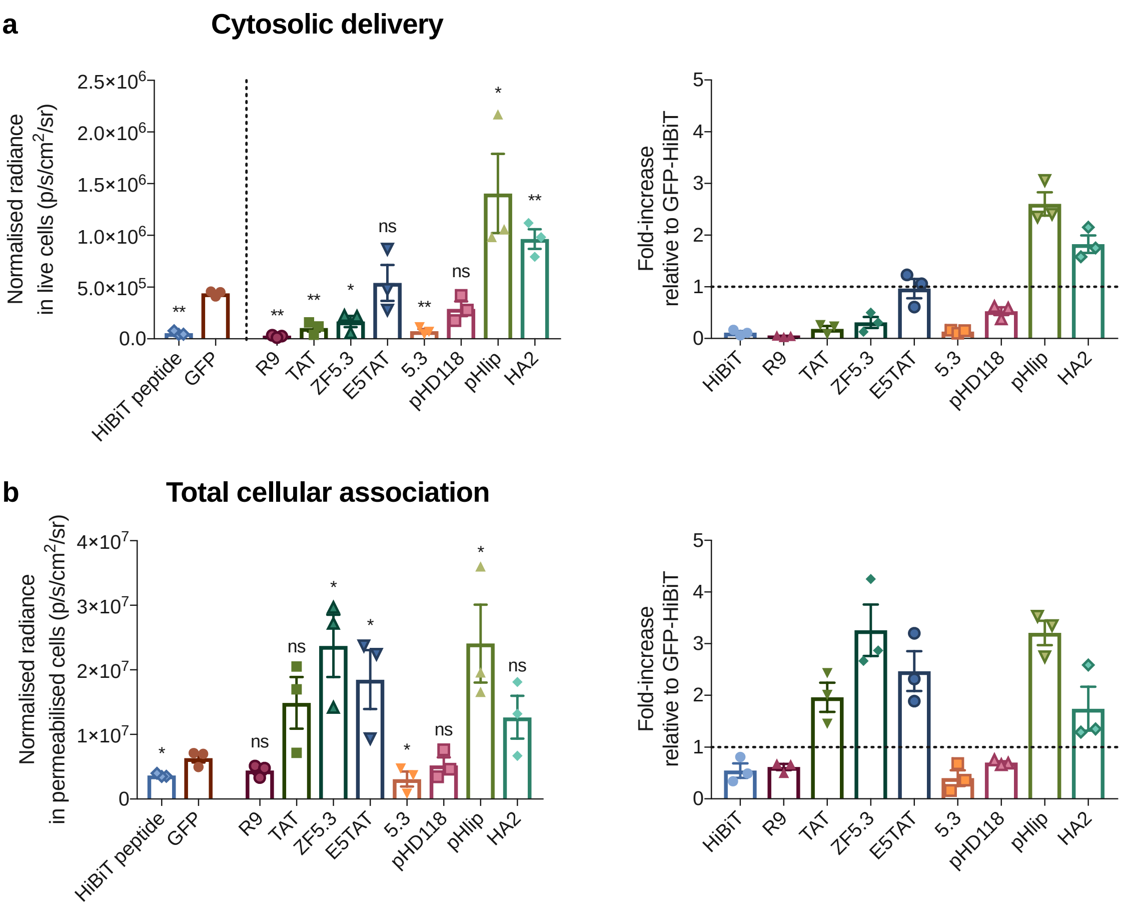

c

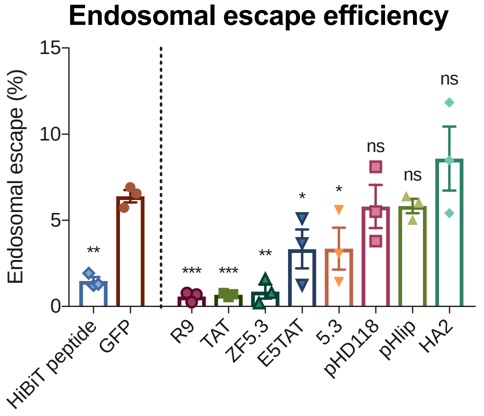

d

Protein	Cytosolic luminescence (x10^{-5}) (p/s/cm^2/sr)	Total cellular association luminescence (x10^{-5}) (p/s/cm^2/sr)	Endosomal escape efficiency (%)
HiBiT	0.55 ± 0.11	36.6 ± 1.7	1.48 ± 0.23
GFP	4.40 ± 0.12	63.6 ± 5.5	6.40 ± 0.27
R9	0.25 + 0.07	44.0 ± 5.5	0.58 ± 0.16
TAT	1.06 ± 0.36	149 ± 40	0.68 ± 0.07
ZF5.3	1.68 ± 0.54	237 ± 48	0.87 ± 0.41
E5TAT	5.41 ± 1.73	185 ± 46	3.34 ± 1.13
5.3	0.75 ± 0.20	30.7 ± 11.7	3.35 ± 1.21
pHD118	2.91 ± 0.71	52.2 ± 12.5	5.81 ± 1.25
pHlip	14.1 ± 3.8	241 ± 60	5.83 ± 0.41
HA2	9.65 ± 0.95	123 ± 33	8.58 ± 1.86

Supplementary Fig. 10: EEPs do not increase endosomal escape efficiency of GFP in HeLa cells. a) Cytosolic luminescent signal of EEP-GFP-HiBiT in HeLa-LSA cells and fold-increase in signal with respect to GFP (represented by dotted line = 1). HeLa-LSA cells were incubated with EEP-GFP-HiBiT proteins at varying concentrations for 4 hours. Data represents mean ± SEM, n=3 independent experiments. Kruskal-Wallis with uncorrected Dunn's test was used to analyse the data. b) Total cellular association of EEP-GFP-HiBiT in HeLa cells and fold-increase with respect to GFP (represented by dotted line = 1) determined by permeabilising the cells using 0.01% w/v digitonin. Data represents mean ± SEM, n=3 independent experiments. Kruskal-Wallis with uncorrected Dunn's test was used to analyse the data. c) Endosomal escape efficiency of EEP-GFP-HiBiT proteins determined by ratioing cytosolic signal with total cellular association. Data represents mean ± SEM, n=3 independent experiments. Kruskal-Wallis with uncorrected Dunn's test was used to analyse the data. d) Summary of cytosolic luminescence, total cellular association luminescence and

endosomal escape efficiency for all proteins. Data represents mean ± SEM, n=3 independent experiments. Kruskal-Wallis with uncorrected Dunn's test was used to analyse the data, with GFP being the control group. ns denote $p > 0.05$, * denotes $p < 0.05$, ** denotes $p < 0.01$ and *** denotes $p < 0.001$

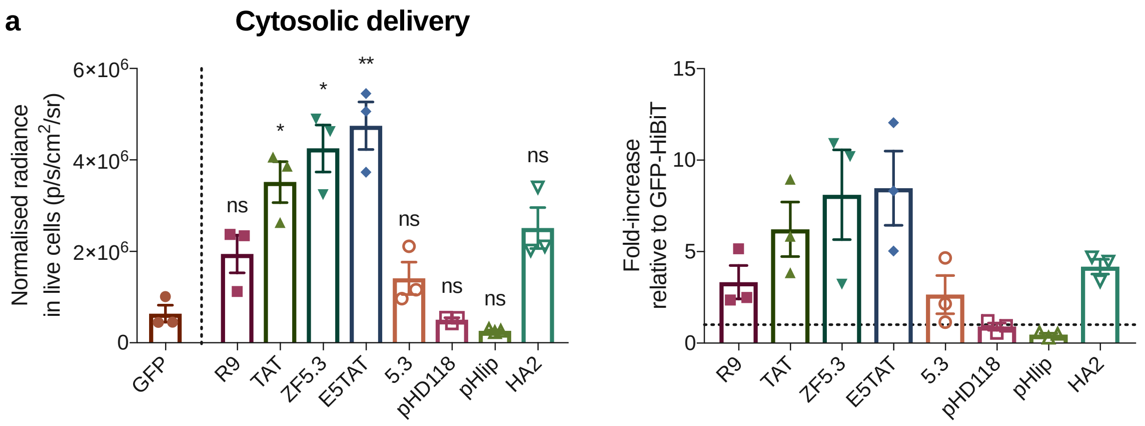

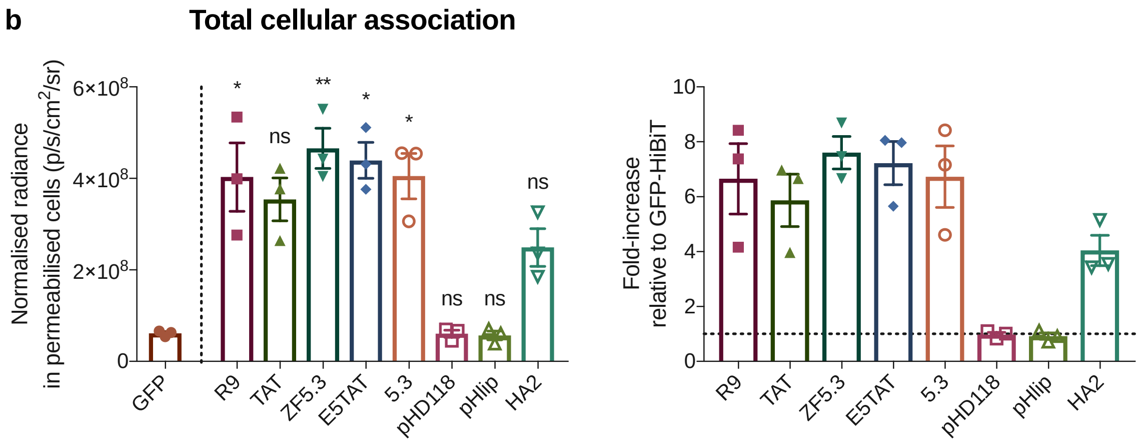

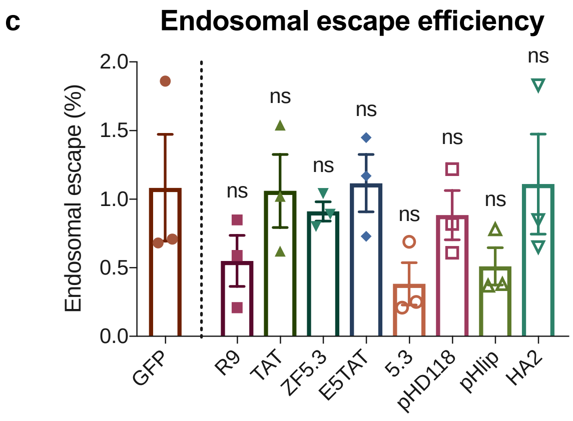

d

Protein	Cytosolic luminescence ($x10^{-6}$) (p/s/cm²/sr)	Total cellular association luminescence ($x10^{-6}$) (p/s/cm²/sr)	Endosomal escape efficiency (%)
GFP	0.64 ± 0.19	61.3 ± 3.7	1.08 ± 0.39
R9	1.94 ± 0.41	403 ± 74	0.55 ± 0.19
TAT	3.51 ± 0.45	355 ± 47	1.06 ± 0.26
ZF5.3	4.25 ± 0.51	466 ± 44	0.91 ± 0.07
E5TAT	4.75± 0.52	439 ± 39	1.12 ± 0.21
5.3	1.41 ± 0.35	405 ± 49	0.39 ± 0.15
pHD118	0.51 ± 0.04	60.6 ± 7.8	0.88 ± 0.18
pHlip	0.26 ± 0.02	56.8 ± 9.5	0.51 ± 0.13
HA2	2.51 ± 0.45	249 ± 41	1.11 ± 0.37

Supplementary Fig. 11: EEPs do not increase endosomal escape efficiency of GFP in a concentration-dependent manner in HEK293 cells. a) Cytosolic luminescent signal of EEP-GFP-HiBiT in HEK293-LSA cells and fold-increase in signal with respect to GFP (represented by dotted line = 1). HEK293-LSA cells were incubated with EEP-GFP-HiBiT proteins at 10 µM for 4 hours. Data represents mean ± SEM, n=3 independent experiments. Kruskal-Wallis with uncorrected Dunn's test was used to analyse the data. b) Total cellular association of EEP-GFP-HiBiT in HEK293 cells and fold-increase with respect to GFP (represented by dotted line = 1) determined by permeabilising the cells using 0.01% w/v

digitonin. Data represents mean ± SEM, n=3 independent experiments. Kruskal-Wallis with uncorrected Dunn's test was used to analyse the data. c) Endosomal escape efficiency of EEP-GFP-HiBiT proteins determined by ratioing cytosolic signal with total cellular association. Data represents mean ± SEM, n=3 independent experiments. Kruskal-Wallis with uncorrected Dunn's test was used to analyse the data. d) Summary of cytosolic luminescence, total cellular association luminescence and endosomal escape efficiency for all proteins. Data represents mean ± SEM, n=3 independent experiments. Kruskal-Wallis with uncorrected Dunn's test was used to analyse the data, with GFP being the control group. ns denotes $p > 0.05$, * denotes $p < 0.05$ and ** denotes $p < 0.01$

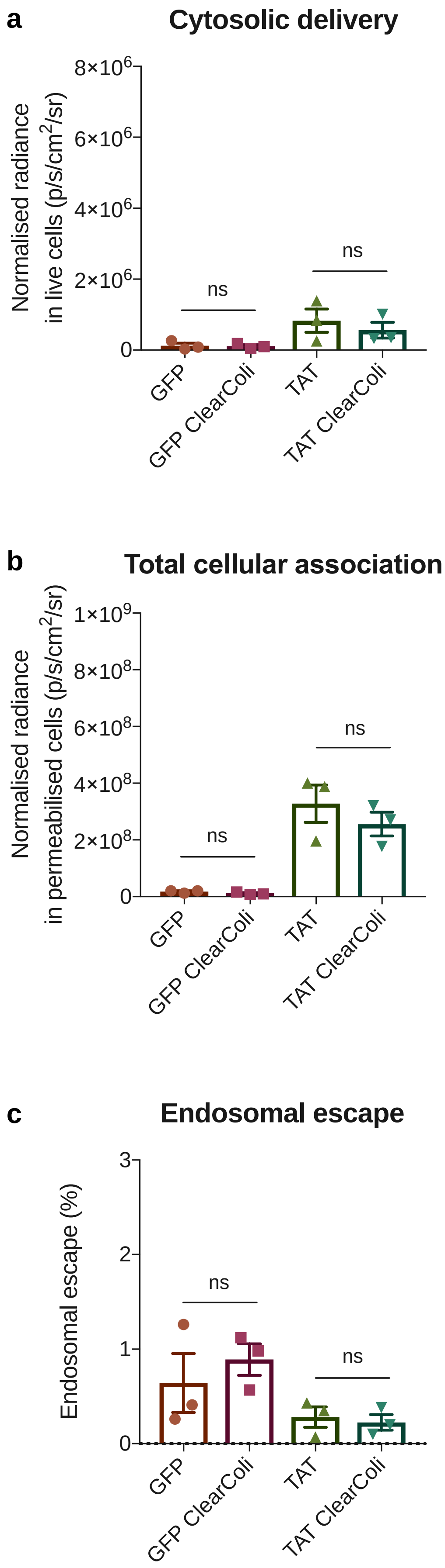
a
Cytosolic delivery
Normalised radiance in live cells (p/s/cm²/sr)
8×10⁶
6×10⁶
4×10⁶
2×10⁶
0
ns
ns
GFP
GFP ClearColi
TAT
TAT ClearColi
b
Total cellular association
Normalised radiance in permeabilised cells (p/s/cm²/sr)
1×10⁹
8×10⁸
6×10⁸
4×10⁸
2×10⁸
0
ns
ns
GFP
GFP ClearColi
TAT
TAT ClearColi
c
Endosomal escape
Endosomal escape (%)
3
2
1
0
ns
ns
GFP
GFP ClearColi
TAT
TAT ClearColi

Supplementary Fig. 12: Endosomal escape efficiency of GFP-HiBiT and TAT-GFP-HiBiT synthesised in E. Coli or ClearColi remains similar. a) Cytosolic luminescent signal of GFP-HiBiT and TAT-GFP-HiBiT in HEK293-LSA cells. HEK293-LSA cells were incubated with proteins at 1 µM for 4 hours. Two-tailed unpaired t test was used to analyse the data. GFP was compared to GFP ClearColi, TAT was compared to TAT ClearColi. Data represents mean ± SEM, n=3 independent experiments. b) Total cellular association in HEK293-LSA cells determined by permeabilising the cells using 0.01% w/v digitonin. Two-tailed unpaired t test was used to analyse the data. GFP was compared to GFP ClearColi, TAT was compared to TAT ClearColi. Data represents mean ± SEM, n=3 independent experiments. c) Endosomal escape efficiency proteins determined by ratioing cytosolic signal with total cellular association. Two-tailed unpaired t test was used to analyse the data. GFP was compared to GFP ClearColi, TAT was compared to TAT ClearColi. Data represents mean ± SEM, n=3 independent experiments. ns (not significant) denotes $p > 0.05$

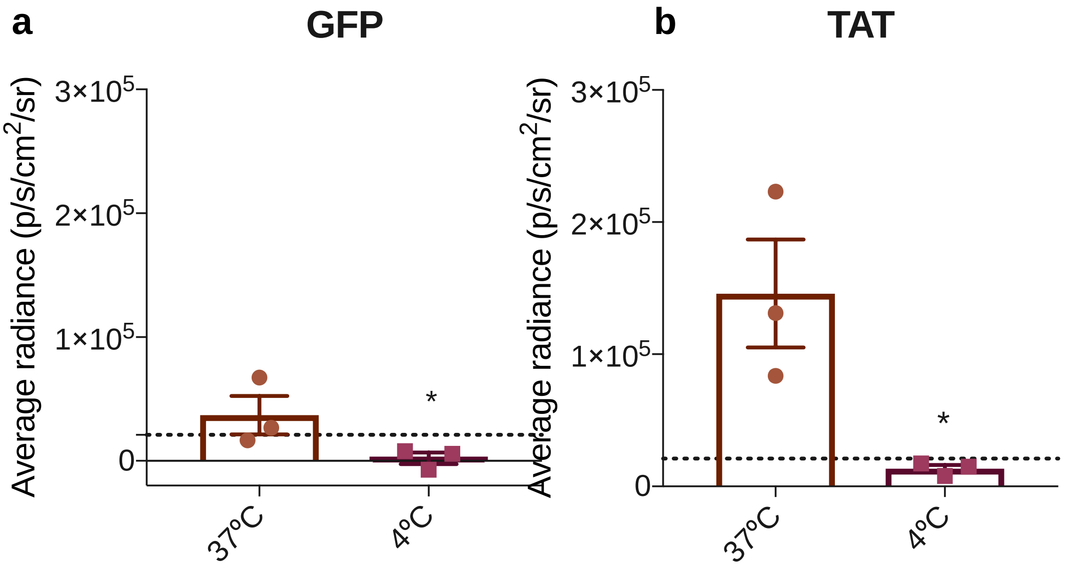

Supplementary Fig. 13: Incubation at 4ºC reduces cytosolic delivery of proteins. HEK293-LSA cells were treated with 1 µM of a) GFP-HiBiT or b) TAT-GFP-HiBiT at 37ºC and 4ºC for 4 hours to compare their level of cytosolic delivery. Dotted line indicates limit of detection. One-tailed unpaired t test was used to analyse this data. Data represents mean ± SEM, n=3 independent experiments. ns denotes $p > 0.05$ and * denotes $p < 0.05$

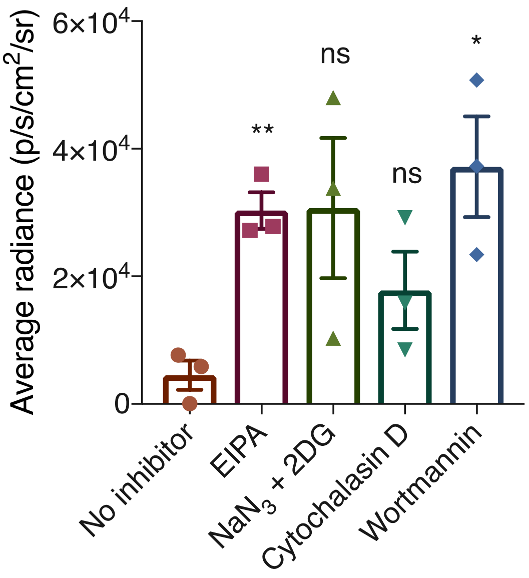

Supplementary Fig. 14: Luminescent signal in cell supernatant after treatment with endocytosis inhibitors. HEK293-LSA cells were treated with endocytosis inhibitors and 1 µM of GFP-HiBiT. After 4-hour incubation, luminescence in cell supernatant was measured. Two-tailed unpaired t test was use to compare between no inhibitor and inhibitor treatment. Data represents mean ± SEM, n=3 independent experiments. ns denotes $p > 0.05$, * denotes $p < 0.05$ and ** denotes $p < 0.01$

GFP

MSKHHHHSGHHHTGHHHHSGSHHHTGHINLKVKGQDGNEVFFRIKRSTQLKKLMNAYCD
RQSVDMTAIAFLFDGRRLRAEQTPDELEMEDGDEIDAMLHQTGGSKGEELFTGVVPILVELDG
DVNGHKFSVRGEGEGDATNGKLTLKFICTTGKLPVPWPTLVTTLTYGVLCFSRYPDHMKRHDF
FKSAMPEGYVQERTISFKDDGTYKTRAEVKFEGDTLVNRIELKGIDFKEDGNILGHKLEYNFNSH
NVYITADKQKNGIKAYFKIRHNVEDGSVQLADHYQQNTPIGDGPVLLPDNHYLSTQSVLSKD
PNEKRDHMVLLEDVTAAGITHGMDELYKVSGWRLFKKISGGSG

R9

MSKHHHHSGHHHTGHHHHSGSHHHTGHINLKVKGQDGNEVFFRIKRSTQLKKLMNAYCD
RQSVDMTAIAFLFDGRRLRAEQTPDELEMEDGDEIDAMLHQTGGGRRRRRRRRRSKGEELFT
GVVPILVELDGDVNGHKFSVRGEGEGDATNGKLTLKFICTTGKLPVPWPTLVTTLTYGVLCFSR
YPDHMKRHDFFKSAMPEGYVQERTISFKDDGTYKTRAEVKFEGDTLVNRIELKGIDFKEDGNIL
GHKLEYNFNSHNVYITADKQKNGIKAYFKIRHNVEDGSVQLADHYQQNTPIGDGPVLLPDN
HYLSTQSVLSKDPNEKRDHMVLLEDVTAAGITHGMDELYKVSGWRLFKKISGGSG

TAT

MSKHHHHSGHHHTGHHHHSGSHHHTGHINLKVKGQDGNEVFFRIKRSTQLKKLMNAYCD
RQSVDMTAIAFLFDGRRLRAEQTPDELEMEDGDEIDAMLHQTGGGRKKRRQRRRPPQASSK
GEELFTGVVPILVELDGDVNGHKFSVRGEGEGDATNGKLTLKFICTTGKLPVPWPTLVTTLTYG
VLCFSRYPDHMKRHDFFKSAMPEGYVQERTISFKDDGTYKTRAEVKFEGDTLVNRIELKGIDFK
EDGNILGHKLEYNFNSHNVYITADKQKNGIKAYFKIRHNVEDGSVQLADHYQQNTPIGDGPV
LLPDNHYLSTQSVLSKDPNEKRDHMVLLEDVTAAGITHGMDELYKVSGWRLFKKISGGSG

ZF5.3

MSKHHHHSGHHHTGHHHHSGSHHHTGHINLKVKGQDGNEVFFRIKRSTQLKKLMNAYCD
RQSVDMTAIAFLFDGRRLRAEQTPDELEMEDGDEIDAMLHQTGGWYSCNVCGKAFVLSRHL
NRHLRVHRRATASSKGEELFTGVVPILVELDGDVNGHKFSVRGEGEGDATNGKLTLKFICTTGK
LPVPWPTLVTTLTYGVLCFSRYPDHMKRHDFFKSAMPEGYVQERTISFKDDGTYKTRAEVKFE
GDTLVNRIELKGIDFKEDGNILGHKLEYNFNSHNVYITADKQKNGIKAYFKIRHNVEDGSVQLA
DHYQQNTPIGDGPVLLPDNHYLSTQSVLSKDPNEKRDHMVLLEDVTAAGITHGMDELYKVS
GWRLFKKISGGSG

E5TAT

MSKHHHHSGHHHTGHHHHSGSHHHTGHINLKVKGQDGNEVFFRIKRSTQLKKLMNAYCD
RQSVDMTAIAFLFDGRRLRAEQTPDELEMEDGDEIDAMLHQTGGGLFEAIAEFIENGWEGLIE
GMGRKKRRQRRRPPQASSKGEELFTGVVPILVELDGDVNGHKFSVRGEGEGDATNGKLTLKFI
CTTGKLPVPWPTLVTTLTYGVLCFSRYPDHMKRHDFFKSAMPEGYVQERTISFKDDGTYKTRA
EVKFEGDTLVNRIELKGIDFKEDGNILGHKLEYNFNSHNVYITADKQKNGIKAYFKIRHNVEDG
SVQLADHYQQNTPIGDGPVLLPDNHYLSTQSVLSKDPNEKRDHMVLLEDVTAAGITHGMDE
LYKVSGWRLFKKISGGSG

5.3

MSKHHHHSGHHHTGHHHHSGSHHHTGHINLKVKGQDGNEVFFRIKRSTQLKKLMNAYCD
RQSVDMTAIAFLFDGRRLRAEQTPDELEMEDGDEIDAMLHQTGGGPSQPTYPGDDAPVRDLI
RFYRDLRRYLNVVTRHRYASSKGEELFTGVVPILVELDGDVNGHKFSVRGEGEGDATNGKLTLK
FICTTGKLPVPWPTLVTTLTYGVLCFSRYPDHMKRHDFFKSAMPEGYVQERTISFKDDGTYKTR
AEVKFEGDTLVNRIELKGIDFKEDGNILGHKLEYNFNSHNVYITADKQKNGIKAYFKIRHNVED
GSVQLADHYQQNTPIGDGPVLLPDNHYLSTQSVLSKDPNEKRDHMVLLEDVTAAGITHGMD
ELYKVSGWRLFKKISGGSG

pHD118

MSKHHHHSGHHHTGHHHHSGSHHHTGHINLKVKGQDGNEVFFRIKRSTQLKKLMNAYCD
RQSVDMTAIAFLFDGRRLRAEQTPDELEMEDGDEIDAMLHQTGGIGEVLHELADDLPELQSWI
KAAQQLSKGEELFTGVVPILVELDGDVNGHKFSVRGEGEGDATNGKLTLKFICTTGKLPVPWPT
LVTTLTYGVLCFSRYPDHMKRHDFFKSAMPEGYVQERTISFKDDGTYKTRAEVKFEGDTLVNRI
ELKGIDFKEDGNILGHKLEYNFNSHNVYITADKQKNGIKAYFKIRHNVEDGSVQLADHYQQNT
PIGDGPVLLPDNHYLSTQSVLSKDPNEKRDHMVLLEDVTAAGITHGMDELYKVSGWRLFKKIS
GGSG

pHlip

MSKHHHHSGHHHTGHHHHSGSHHHTGHINLKVKGQDGNEVFFRIKRSTQLKKLMNAYCD
RQSVDMTAIAFLFDGRRLRAEQTPDELEMEDGDEIDAMLHQTGGGRKKRRQRRRPPQASSK

GEELFTGVVPILVELDGDVNGHKFSVRGEGEGDATNGKLTLKFICTTGKLPVPWPTLVTTLTYG
VLCFSRYPDHMKRHDFFKSAMPEGYVQERTISFKDDGTYKTRAEVKFEGDTLVNRIELKGIDFK
EDGNILGHKLEYNFNSHNVYITADKQKNGIKAYFKIRHNVEDGSVQLADHYQQNTPIGDGPV
LLPDNHYLSTQSVLSKDPNEKRDHMVLLEDVTAAGITHGMDELYKVSGWRLFKKISGGSG

HA2

MSKHHHHSGHHHTGHHHHSGSHHHTGHINLKVKGQDGNEVFFRIKRSTQLKKLMNAYCD
RQSVDMTAIAFLFDGRRLRAEQTPDELEMEDGDEIDAMLHQTGGGLFEAIEGFIENGWEGMI
DGWYGASSKGEELFTGVVPILVELDGDVNGHKFSVRGEGEGDATNGKLTLKFICTTGKLPVPW
PTLVTTLTYGVLCFSRYPDHMKRHDFFKSAMPEGYVQERTISFKDDGTYKTRAEVKFEGDTLVN
RIELKGIDFKEDGNILGHKLEYNFNSHNVYITADKQKNGIKAYFKIRHNVEDGSVQLADHYQQ
NTPIGDGPVLLPDNHYLSTQSVLSKDPNEKRDHMVLLEDVTAAGITHGMDELYKVSGWRLFK
KISGGSG

Supplementary Fig. 15: Full sequences of uncleaved protein samples used in this study. Sequences are coded as following: grey = 14xHis purification tag and bdSUMO; blue and underlined = endosomal escape peptide sequence; green = muGFP; yellow = HiBiT; none = amino acids intended to protect HiBiT peptide from C-terminal degradation. Note: bdSENP1 cleaves after bdSUMO C-terminal GG.

Name	Sequence (5' – 3')	Purpose
**Constructs: pET – bdSUMO – EEP – GFP - HiBit**		
P1	GGTACCACGCGTGCGCGCTGATCCGG	Forward primer to generate vector PCR fragment to gibson assemble with 14xHis – bdSUMO – E5TAT – GFP – HiBit gene fragment.
P2	ATGTATATCTCCTTCTTAAAGTTAAACAAAAT	Reverse primer to generate vector PCR fragment to gibson assemble with 14xHis – bdSUMO – E5TAT – GFP – HiBit gene fragment.
P3	CATCTTTGAAAGATATAGTGCGTTCCT	Reverse primer to generate all EEP PCR fragments.
P4	GAGATTGACGCCATGTTACACCAGACCGGTGGGAGCAAAGGAGAAGAACTTTTCACTG	Forward primer to generate GFP only PCR fragment.
P5	GAGATTGACGCCATGTTACACCAGACCGGTGGGGGTCGTAAAAAACGTCGTCAG	Forward primer to generate TAT PCR fragment.
P6	GAGATTGACGCCATGTTACACCAGACCGGTGGGGGTCGTAGAAGACGGCGGCG	Forward primer to generate R9 PCR fragment.
P7	GAGATTGACGCCATGTTACACCAGACCGGTGGGTGGTACTCTTGCAACGTTTGCG	Forward primer to generate ZF5.3 PCR fragment.
P8	GAGATTGACGCCATGTTACACCAGACCGGTGGGGGTCCGTCTCAGCCGACC	Forward primer to generate 5.3 PCR fragment.
P9	GAGATTGACGCCATGTTACACCAGACCGGTGGGATCGGCGAAGTGCTGC	Forward primer to generate pHD118 PCR fragment.
P10	GAGATTGACGCCATGTTACACCAGACCGGTGGGGCTGAGGAGCAACAACCATGG	Forward primer to generate pHlip PCR fragment.
P11	GAGATTGACGCCATGTTACACCAGACCGGTGGGGGTCTGTTCGAAGCGATCG	Forward primer to generate HA2 PCR fragment.

**Construct: pCDH - Large BiT**		
P12	TAGAGCTAGCGAATTCGATGGTCTTCACACTCGAAG	Forward primer to amplify Large BiT with pCDH vector overhangs.
P13	GTACTGAGGATCCCTAACTGTTGATGGTTACTCGGAAC	Reverse primer to amplify Large BiT with pCDH vector overhangs.
**Construct: pCDH - Large BiT - SNAPtag- B-Actin**		
P14	GACCGGCGCCTACTCTAGAGGCCACCATGATGGTCTTCACACTCGAAGA	Forward primer to amplify Large BiT with pCDH overhang.
P15	ATTTCGCAGTCTTTGTCCATACTGTTGATGGTTACTCGGA	Reverse primer to amplify Large BiT with SNAP-tag overhang.
P16	TCCGAGTAACCATCAACAGTATGGACAAAGACTGCGAAAT	Forward primer to amplify SNAP-tag with Large BiT overhang.
P17	CGGCGATATCATCATCCATGGATCCGCCTGCAGGACCCAG	Reverse primer to amplify SNAP-tag with □-Actin overhang.
P18	ATGGATGATGATATCGCCGCGCTC	Forward primer to amplify □-Actin.
P19	GATCCGATTTAAATTCGAATTCCTAGAAGCATTTGCGGTGGACG	Reverse primer to amplify □-Actin with pCDH overhang.
**Construct: pET - LargeBit**		
P20	GAAAACCTGTACTTCCAATCCGGTT	Forward primer to generate pET vector PCR fragment for assembly with LargeBit fragment.
P21	CATATGTATATCTCCTTCTTAAAGTTAAACAA	Reverse primer to generate pET vector PCR fragment for assembly with LargeBit fragment.
P22	TTGTTTAACTTTAAGAAGGAGATATACATATGGTCTTCACACTCGAAGATTTCG	Forward primer to generate LargeBit PCR fragment with vector overhangs.
P23	AACCGGATTGGAAGTACAGGTTTTCACTAGTACTGTTGATGGTTACTCGGAAC	Reverse primer to generate LargeBit PCR fragment with vector overhangs.

Supplementary Table 1: Complete list of primers used.

BSA	Expected average mass (kDa)	Observed mass (M/Z)
**$[M+H]^+$**	~ 66.5	66777.4
**$[M+2H]^{+2}$**	~ 33.3	33255.6

Supplementary Table 2: Calibration of the MALDI instrument was performed with BSA. Observed masses are peak centroid values, expected masses were observed within the peak which is relatively broad due to isotopic combinations.

	GFP	R9	TAT	ZF5.3	E5TAT	5.3	pHD118	pHlip	HA2
**Expected average mass $[M+H]^+$ (Da)**	28212.9	29675.6	30072	31733.9	32379.6	32716.9	31012	31200.2	30943.8
**Observed mass (M/Z)**	**28075.8**	**29524.9**	**29936.2**	**31587.0**	**32227.7**	**32578.4**	**31370.6**	**31058.5**	**30811.9**
**Minor Fragments (corresponding amino acids lost from C-terminus)**			*29478.0 (-ISGGSG)*	*31135.6 (-ISGGSG)*		*31865.9 (-KKISGGSG)*		*30595.0 (-ISGGSG)*	
			29222.2 (-KKISGGSG)	*30869.5 (-KKISGGSG)*				*30467.7 (-KISGGSG)*	
				30732.1 (-FKKISGGSG)				*30347.1 (-KKISGGSG)*	
								30195.0 (FKKISGGSG)	
								30084.4 (-LFKKISGGSG)	

Supplementary Table 3: MALDI mass spectroscopy peak analysis of EEP-GFP-HiBiT proteins. Observed masses are peak centroid values. Expected masses were observed within the peak which is relatively broad due to isotopic combinations. This is consistent with the difference in mass observed for BSA (Supplementary Table 1). The unusually broad appearance of pHD118 results in a centroid peak value that is greater than all other proteins, however the expected average mass is found within this broad peak. The minor fragments observed for some proteins correlate with a sequential loss of amino acids from the C-terminus, which is expected to be laser induced. Any true truncation which could affect the activity of HiBiT is compensated for by measuring the relative activity of each protein (Fig. 3).

Peptide sequence	Mass of fragment $[M+H]^+$
LFKKISGGSG	976.2
FKKISGGSG	863.0
KKISGGSG	715.8
KISGGSG	587.6
ISGGSG	459.5

Supplementary Table 4: Sequential peptide fragments from the C-terminus of EEP-GFP-HiBiT proteins which correlate with fragments observed in Supplementary Table 3.
