# Supplementary material for: Unravelling cytosolic delivery of cell penetrating peptides with a quantitative endosomal escape assay
Source: Nat Commun. 2021 Jun 17;12:3721. doi: 10.1038/s41467-021-23997-x (PMC8211857; doi:10.1038/s41467-021-23997-x)
Supplement: Supplementary file 2 — Reporting Summary [file 41467_2021_23997_MOESM2_ESM.pdf]

nature research

Corresponding author(s): Angus P.R. Johnston & Colin W. Pouton

Last updated by author(s): May 13, 2021

# Reporting Summary

Nature Research wishes to improve the reproducibility of the work that we publish. This form provides structure for consistency and transparency in reporting. For further information on Nature Research policies, see Authors & Referees and the Editorial Policy Checklist .

## Statistics

For all statistical analyses, confirm that the following items are present in the figure legend, table legend, main text, or Methods section.

| n/a | Confirmed | |
|---|---|---|
| ☐ | ☒ | The exact sample size (*n*) for each experimental group/condition, given as a discrete number and unit of measurement |
| ☐ | ☒ | A statement on whether measurements were taken from distinct samples or whether the same sample was measured repeatedly |
| ☐ | ☒ | The statistical test(s) used AND whether they are one- or two-sided<br>*Only common tests should be described solely by name; describe more complex techniques in the Methods section.* |
| ☒ | ☐ | A description of all covariates tested |
| ☐ | ☒ | A description of any assumptions or corrections, such as tests of normality and adjustment for multiple comparisons |
| ☐ | ☒ | A full description of the statistical parameters including central tendency (e.g. means) or other basic estimates (e.g. regression coefficient) AND variation (e.g. standard deviation) or associated estimates of uncertainty (e.g. confidence intervals) |
| ☐ | ☒ | For null hypothesis testing, the test statistic (e.g. *F*, *t*, *r*) with confidence intervals, effect sizes, degrees of freedom and *P* value noted<br>*Give P values as exact values whenever suitable.* |
| ☒ | ☐ | For Bayesian analysis, information on the choice of priors and Markov chain Monte Carlo settings |
| ☒ | ☐ | For hierarchical and complex designs, identification of the appropriate level for tests and full reporting of outcomes |
| ☒ | ☐ | Estimates of effect sizes (e.g. Cohen's *d*, Pearson's *r*), indicating how they were calculated |

*Our web collection on statistics for biologists contains articles on many of the points above.*

## Software and code

Policy information about availability of computer code

| | |
|---|---|
| Data collection | Luminescence data was acquired using Living Image 4.3.1. Fluorescence images were obtained with SlideBook 6.0 and Leica LAS X. CellCapture 4.0 was used to acquire flow cytometry data. |
| Data analysis | Luminescence data was processed using Living Image 4.3.1. GraphPad Prism 7 was used for plotting graphs and statistical analyses. SlideBook 6.0 and ImageJ 2.0.0 was used for fluorescence image analysis. FlowJo 10 was used to analyse flow cytometry data. mMass 5.4.0 was used to analyse MALDI spectra. |

For manuscripts utilizing custom algorithms or software that are central to the research but not yet described in published literature, software must be made available to editors/reviewers. We strongly encourage code deposition in a community repository (e.g. GitHub). See the Nature Research guidelines for submitting code & software for further information.

## Data

Policy information about availability of data

All manuscripts must include a data availability statement. This statement should provide the following information, where applicable:

- Accession codes, unique identifiers, or web links for publicly available datasets
- A list of figures that have associated raw data
- A description of any restrictions on data availability

The data that support the findings of this study are available from the corresponding author upon reasonable request.

# Field-specific reporting

Please select the one below that is the best fit for your research. If you are not sure, read the appropriate sections before making your selection.

## Life sciences study design

All studies must disclose on these points even when the disclosure is negative.

| | |
|---|---|
| Sample size | No sample-size calculation was performed. A sample size of 3 was determined to be adequate as the statistical analysis showed significant differences as noted in the manuscript. |
| Data exclusions | No data was excluded from the analyses. |
| Replication | All experiments were performed at least three times independently for the figures in the main manuscript unless otherwise noted. All attempts at replication were successful. |
| Randomization | For luminescence measurements, the well position and order in which samples are added to cells were randomised. For all other experiments, the order of sample analysis was randomised. |
| Blinding | Blinding is not relevant to this study as we are comparing endosomal escape efficiencies of different peptides across different cell lines. There was no preconception for which sample would have higher signal. |

## Reporting for specific materials, systems and methods

We require information from authors about some types of materials, experimental systems and methods used in many studies. Here, indicate whether each material, system or method listed is relevant to your study. If you are not sure if a list item applies to your research, read the appropriate section before selecting a response.

### Materials & experimental systems

| n/a | Involved in the study | |
|---|---|---|
| ☒ | ☐ | Antibodies |
| ☐ | ☒ | Eukaryotic cell lines |
| ☒ | ☐ | Palaeontology |
| ☒ | ☐ | Animals and other organisms |
| ☒ | ☐ | Human research participants |
| ☒ | ☐ | Clinical data |

### Methods

| n/a | Involved in the study | |
|---|---|---|
| ☒ | ☐ | ChIP-seq |
| ☒ | ☐ | Flow cytometry |
| ☒ | ☐ | MRI-based neuroimaging |

## Eukaryotic cell lines

Policy information about cell lines

| | |
|---|---|
| Cell line source(s) | HEK293 cells were sourced from ATCC and Hela cells were a kind gift from Prof David Jans, Monash University (sourced from ATCC). |
| Authentication | No authentication was performed as the cells came from a reliable source. |
| Mycoplasma contamination | All cell lines were tested monthly for mycoplasma contamination by PCR. All cell lines were negative for mycoplasma. |
| Commonly misidentified lines (See ICLAC register) | No commonly misidentified cell lines were used. |
